# Disrupted White Matter Topology Organization in Preschool Children with Tetralogy of Fallot

**DOI:** 10.1002/brb3.70153

**Published:** 2024-11-22

**Authors:** Yuting Liu, Liang Hu, Meijiao Zhu, Jingjing Zhong, Mingcui Fu, Mingwen Yang, Shuting Cheng, Ying Wang, Xuming Mo, Ming Yang

**Affiliations:** ^1^ Department of Radiology Children's Hospital of Nanjing Medical University Nanjing Jiangsu Province China; ^2^ Department of Cardiothoracic Surgery Children's Hospital of Nanjing Medical University Nanjing Jiangsu Province China

**Keywords:** brain structural network, congenital heart disease, cognitive impairment, diffusion tensor imaging, tetralogy of fallot

## Abstract

**Background**: Cognitive impairment is the most common long‐term complication in children with congenital heart disease (CHD) and is closely related to the brain network. However, little is known about the impact of CHD on brain network organization. This study aims to investigate brain structural network properties that may underpin cognitive deficits observed in children with Tetralogy of Fallot (TOF).

**Methods**: In this prospective study, 29 preschool‐aged children diagnosed with TOF and 19 without CHD (non‐CHD) were enrolled. Participants underwent diffusion tensor imaging (DTI) scans alongside cognitive assessment using the Chinese version of the Wechsler Preschool and Primary Scale of Intelligence—fourth edition (WPPSI‐IV). We constructed a brain structural network based on DTI and applied graph analysis methodology to investigate alterations in diverse network topological properties in TOF compared with non‐CHD. Additionally, we explored the correlation between brain network topology and cognitive performance in TOF.

**Results**: Although both TOF and non‐CHD exhibited small‐world characteristics in their brain networks, children with TOF significantly demonstrated increased characteristic path length and decreased clustering coefficient, global efficiency, and local efficiency compared with non‐CHD (*p* < 0.05). Regionally, reduced nodal betweenness and degree were found in the left cingulate gyrus, and nodal efficiency was decreased in the right precentral gyrus and cingulate gyrus, left inferior frontal gyrus (triangular part), and insula (*p* < 0.05). Furthermore, a positive correlation was identified between local efficiency and cognitive performance (*p* < 0.05).

**Conclusion**: This study elucidates a disrupted brain structural network characterized by impaired integration and segregation in preschool TOF, correlating with cognitive performance. These findings indicated that the brain structural network may be a promising imaging biomarker and potential target for neurobehavioral interventions aimed at improving brain development and preventing lasting impairments across the lifetime.

## Graphical abstract

Schematic diagram of the study. Population enrollment and data collection **(a)**. Construction and analysis of brain structure network **(b)**. The main results of the study **(c)**. TOF = Tetralogy of Fallot; HC = Health control; MRI = Magnetic resonance imaging; WPPSI‐IV = the Wechsler Preschool and Primary Scale of Intelligence—fourth edition.

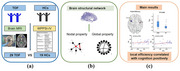

**Abbreviations**
CHD: Congenital Heart Disease
DTI: Diffusion Tensor Imaging
TOF: Tetralogy of Fallot

## 1 Introduction

Congenital heart disease (CHD) is the most common congenital defect in children (van der Linde et al. 2011). Notably, advancements in surgical techniques and perioperative care have augmented survival rates; a growing number of children with CHD have been able to survive to adulthood. However, cognitive impairments encompassing deficits in language, attention, executive function, and memory are widely recognized as predominant long‐term sequelae in this population (Olsen et al. 2011; Sananes et al. 2012; Tabbutt, Gaynor, and Newburger 2012; Naef et al. 2017). These neurodevelopmental disorders may stem from early brain development impairment or acquired brain damage. Compared with non‐cyanotic CHD children, cyanotic CHD children experienced diminished oxygen delivery to various systemic organs, including the brain, potentially leading to delayed brain maturation (Yagi et al. 2017; Mulkey et al. 2014; Kelly et al. 2017). These adverse cognitive performances have spurred investigations into brain development in CHD, particularly the utilization of noninvasive multimodal MRI techniques. Multimodal MRI studies have revealed reduced brain volume, aberrant cortical morphology, white matter microstructural abnormalities, and impaired brain function both preoperatively and postoperatively, some of which exhibit significant associations with unfavorable cognitive performance (Kelly et al. 2017; de Asis‐Cruz et al. 2018; Fontes et al. 2019; Easson et al. 2020; Morton et al. 2020). However, despite these findings, the neurobiological mechanisms underlying cognitive impairment in CHD remain to be fully elucidated.

Brain network organization is closely linked to cognitive deficits and neurological disorders, offering insights into the neuropathophysiological mechanisms of brain diseases and the identification of biomarkers associated with cognitive impairment (Petersen and Sporns 2015; Rubinov and Sporns 2010). Currently, only a limited number of studies have explored brain network organization in CHD, with a focus on mixed‐type CHD or infants and adolescents. Given that anatomical, physiological, and hemodynamic stability profoundly influence brain development (Peyvandi et al. 2019), there is a compelling need to investigate brain developmental patterns in individuals with different types of CHD to delineate their specific neuroimaging biomarkers. However, knowledge regarding the brain structural network underlying cognitive impairments in preschoolers with the subtype of CHD (TOF) remains limited.

Advancements in graph analysis techniques have facilitated the exploration of brain network topology, unveiling crucial insights into network integration, segregation, and small‐worldness (Fair et al. 2007). These metrics serve as reliable biomarkers of brain abnormalities and are related to various neurodevelopment disorders (Shu et al. 2011; Owen et al. 2013), commonly utilized to elucidate the neural mechanisms underlying deficits observed in conditions such as ADHD, autism, and prematurity (Sidlauskaite et al. 2015; Kaku et al. 2019; Sa de Almeida et al. 2021). Drawing from prior research, we hypothesize that children with TOF exhibit disruptions in their brain structural networks and that these disruptions are correlated with poorer cognitive outcomes. To test this hypothesis, we applied graph theory analysis to measure brain structural network topology properties, including global properties and regional properties, using DTI in TOFs and controls. We further investigated whether structural network topology is related to cognitive outcomes.

## 2 Methods

### 2.1 Participants

**FIGURE 1:**
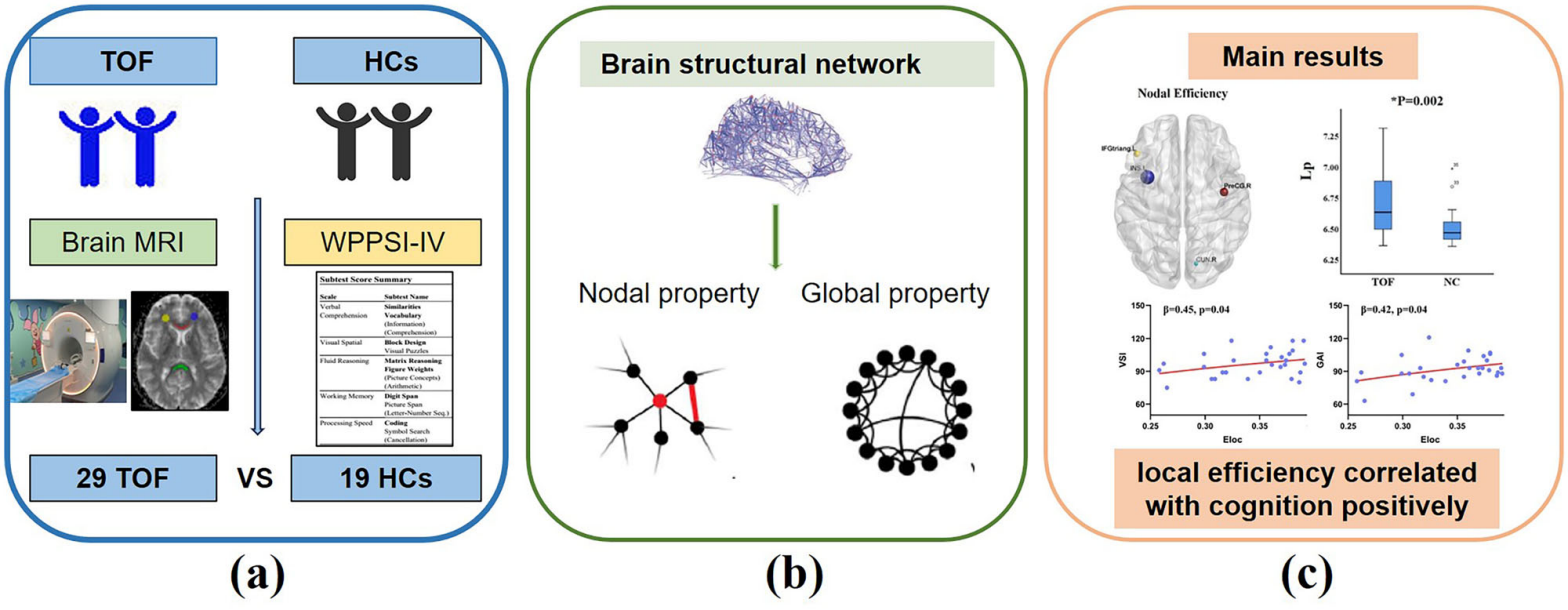
Schematic diagram of the study. Population enrollment and data collection **(a)** Construction and analysis of brain structure network **(b)** The main results of the study **(c)** HC=Health control; MRI=Magnetic resonance imaging; TOF=Tetralogy of Fallot; WPPSI‐IV=the Wechsler Preschool and Primary Scale of Intelligence—fourth edition.

Thirty‐five children diagnosed with TOF undergoing corrective surgery and twenty‐five non‐CHD controls, matched for gender, education, and age, were recruited from the Children's Hospital of Nanjing Medical University between June 2019 and October 2021. All of these children with TOF had one operation. The inclusion and exclusion criteria were detailed in our prior study (Yang et al. 2022). In brief, inclusion criteria for children with TOF were: (1) age ranging from 3–6 years; (2) absence of syndromic conditions, metabolic disorders, or other congenital disease; (3) no history of tumor, trauma, or central nervous system diseases; (4) no prior mental illness or psychiatric medication; (5) cardiopulmonary bypass surgery performed before age 3; and (6) right‐handedness. 25 non‐CHD controls were recruited from: (1) outpatients with transient fever (resolved without health issues at follow‐up lasting more than 6 months); (2) children undergoing routine physical examinations at the child health care clinic; and (3) volunteers from the community. Inclusion criteria for non‐CHD controls were: (1) absence of congenital or metabolic diseases; (2) no history of mental illness or psychiatric medication; (3) no central nervous system diseases; (4) no prior surgical history; and (5) right‐handedness. Four TOF participants were excluded for not meeting the inclusion criteria, and two TOF and six controls were excluded due to excessive head motion. Ultimately, twenty‐nine children with TOF and nineteen children without CHD were included in the final analysis, all without evidence of brain lesions such as infarction or hemorrhage (Figure 1). Written informed consent was obtained from each subject's guardian for data use and review, and the protocol received approval from the Institutional Ethics Committee of Children's Hospital of Nanjing Medical University and consented to participate.

### 2.2 Clinical Variables

The basic demographic and clinical data of children diagnosed with TOF was collected from electronic medical records. This included information such as gender, age at MRI, age at surgery, hospitalization day, operational duration, cardiopulmonary bypass (CPB) time, and aortic cross clamp (ACC) time.

### 2.3 Cognitive Assessment

Cognitive performance was assessed by experienced neuroradiologists one week prior to MRI scans using the Chinese version of the Wechsler Preschool and Primary Scale of Intelligence—fourth edition (WPPSI‐IV) (Jane et al. 2019). Four primary indices were derived, including the verbal comprehension index (VCI), visual spatial index (VSI), working memory index (WMI), and full‐scale intelligence quotient (FSIQ). Additionally, three auxiliary indicators were assessed, comprising the speech reception index (SRI), non‐speech reception index (N‐SRI), and general ability index (GAI), which were also evaluated.

### 2.4 MRI Data Acquisition

All participants underwent brain MRI scanning on a 3.0T MRI system (Ingenia 3.0, Philips Health Care, Best, the Netherlands) using a 16‐channel head coil. MRI scanning was performed at night during natural sleep or with chloral hydrate sedation (1 mL/kg) with parental consent. Earplugs and foam were used to mitigate scanning noise and minimize head motion, respectively. DTI images were obtained using the following parameters: TE = 96 ms, TR = 4618 ms, FOV = 200 × 200 × 140 mm, slice thickness 2 mm, 32 isotropic directions, b‐value = 1000 s/mm2, and acquisition time = 6 min 32 s. Three‐dimensional T1‐weighted high‐resolution structural images were obtained using the following parameters: TE = 3.5 ms, TR = 7.9 ms, FOV = 200 × 200 × 200 mm, slice thickness 1 mm, and acquisition time = 4 min 24 s. Fluid‐attenuated inversion recovery images were acquired to exclude brain lesions using the following parameters: TE = 130 ms, TR = 8500 ms, FOV = 200 × 200 × 119 mm, slice thickness 5 mm, and acquisition time = 2 min 8 s. Subsequently, all images underwent review by two experienced pediatric neuroradiologists, blinded to the details of each participant's medical history. Consensus was reached through discussion when there was a difference of opinion.

### 2.5 MRI Data Preprocessing

All DTI data were preprocessed using PANDA software (Cui et al. 2013.), and white matter structural networks were constructed and analyzed with GRETNA (https://www.nitrc.org/projects/gretna/). The specific steps were as follows: (1) 3D‐T1WI and DTI data were converted from DICOM to NIFTI. (2) Eddy correction was performed to eliminate the effects of head motion and image distortion. (3) The brain structure and tissue extraction. (4) DTI data were co‐registered to 3D‐T1WI, and the co‐registered images were normalized to the Montreal Neurological Institute (MNI) space. (5) FA was obtained in the white matter region based on the JHU white matter tractography atlas.

### 2.6 Brain Structure Network Construction and Analysis

We divided the brain into 90 regions based on the ALL template, with each brain region serving as a node. Deterministic fiber tracking involves the assignment of fibers to specific anatomical bundles based on regions of interest (ROIs) delineated by continuous tracking (FACT) and obtaining structural connectivity between two nodes. Fiber tracking was terminated under conditions of FA < 0.2 in voxel or angle > 45° between two eigenvectors. We considered the mean FA value as the edge and constructed an FA‐weighted 90 × 90 matrix for each participant. We considered structural connectivity to exist only if the fiber numbers between two nodes were > 2.

Global and nodal property analyses were executed using GRETNA (http://www.nitrc.org/projects/gretna/). Global properties of the structural brain network were characterized by the following parameters: Clustering coefficient (CP) is quantified as the percentage of the number of existing connections and the number of maximum possible connections among nodes’ nearest neighbors, which is used to measure network segregation (Watts and Strogatz 1998). Network density (ND) is defined as the average of the total number of connections and is used to measure the overall tightness of the network (Danielle et al. 2017). Characteristic path length (Lp) is defined as the average of the shortest path length that links all nodes, which is used to measure network integration (Rubinov and Sporns 2010). Normalized Cp (γ = Cp real/Cp rand), normalized Lp (λ = Lp real/Lp rand), and small‐worldness (σ = γ/λ) were determined. Global efficiency is defined as the inverse of average shortest path length, which represents the capacity of information transmission over the network, and local efficiency is defined as the average of local efficiency of all individual nodes, which is the measure of fault tolerance of the network (Achard and Bullmore 2007). Compared with regular networks and random networks, a small world network needs to satisfy the following parameters: γ ≫ 1 and λ ≈ 1, and σ > 1, featuring heightened local connectivity and shorter path length (Uehara et al. 2014). The small‐world network, σ, is a fascinating model for the description of complex brain networks that not only support both specialized and integrated information processing but also facilitate an energy‐efficient balance between network segregation and integration (Panigrahy et al. 2015; Yuan, Wade, and Babcock 2015).

Nodal properties were assessed through computation of nodal betweenness, nodal degree, and nodal efficiency. Nodal betweenness quantifies the proportion of the shortest path between two nodes passing through the network and measures the influence of a brain region on network communication. Nodal degree represents the number of edges of a node that directly connect with the remaining nodes in the network, which measures how interactive a node is in the network. Nodal efficiency is the inverse of the harmonic mean of the shortest path length in the network and measures the importance of a node in network communication (Achard and Bullmore 2007).

### 2.7 Statistical Analysis

The Shapiro‐Wilk test was employed to assess the normality of data distribution. Continuous variables are presented as the mean ± standard deviation (m ± sd) or median and range. Unpaired two‐sample t test and chi‐squared test (χ^2^ test) were utilized to analyze the differences in demographic and clinical characteristics between the TOF and non‐CHD groups using SPSS 25.0, with significance level set to *p* < 0.05.

In order to control the potential confounding effects of age and gender, a covariate‐adjusted unpaired two‐sample t test was performed using GRETNA software. Age and gender were incorporated into the model as covariables to assess differences in brain network topology parameters between TOF and non‐CHD. False‐positive correction for node statistical comparison was performed using 1/90 (numbers of node) = 0.0111 as a significance threshold to the multiple comparison correction, which means that nodes with *p* < 0.0111 are significant. This correction method has been used in many studies (Lynall et al. 2010; Meng et al. 2014). Subsequently, employing age and gender as covariates, general linear regression analysis was conducted to investigate the relationship between topological parameters and clinical data and cognitive performance in children with TOF.

## 3 Results

### 3.1 Demographic and Clinical Characteristics

The differences in demographic and clinical data in children with TOF are shown in Table 1. There were no significant differences in gender or age between TOF and controls. There were 15(55.5%), 13(48.1%), 14(51.8%), 15(55.5%), 12(44.4%), 14(51.8%), and 15(55.5%) cognitive outcomes below average in children with TOF.

**TABLE 1:** Demographic data and cognitive performance in TOF and non‐CHD.

Indicators	TOF (*n* = 29)	non‐CHD (*n* = 19)	t/x^2^	*p*
Gender (female/male)	14/15	6/13	1.31	0.25
Age at MRI (years)	4.06 ± 1.18	4.37 ± 0.69	−0.93	0.22
Age at surgery (months, *n* = 27)	12.4 ± 8.5			
Hospitalization days (*n* = 27)	26 (17 ∼ 54)	—	—	
Operational duration (minutes, *n* = 27)	185(135 ∼ 360)	—	—	
CPB (minutes, *n* = 27)	75 (55 ∼ 110)	—	—	
ACC (minutes, *n* = 27)	54 (19.2 ∼ 76)	—	—	
Cognitive assessment				
VCI(*n* = 27)	90.21 ± 15.60	—	—	
VSI(*n* = 27)	96.69 ± 11.54	—	—	
WMI(*n* = 27)	94.72 ± 12.15	—	—	
FSIQ(*n* = 27)	91.90 ± 11.89	—	—	
SRI(*n* = 27)	94.24 ± 16.33	—	—	
N‐SRI(*n* = 27)	94.24 ± 11.45	—	—	
GAI(*n* = 27)	91.90 ± 11.64	—	—	

Abbreviations: ACC, aortic cross‐clamp; CPB, cardiopulmonary bypass; FSIQ, full‐scale intelligence quotient; GAI, general ability index; N‐SRI, non‐speech reception index; SRI, speech reception index; VCI, verbal comprehension index; VSI, visual spatial index; WMI, working memory index.

### 3.2 Whole and Nodal Topology Properties

**FIGURE 2:**
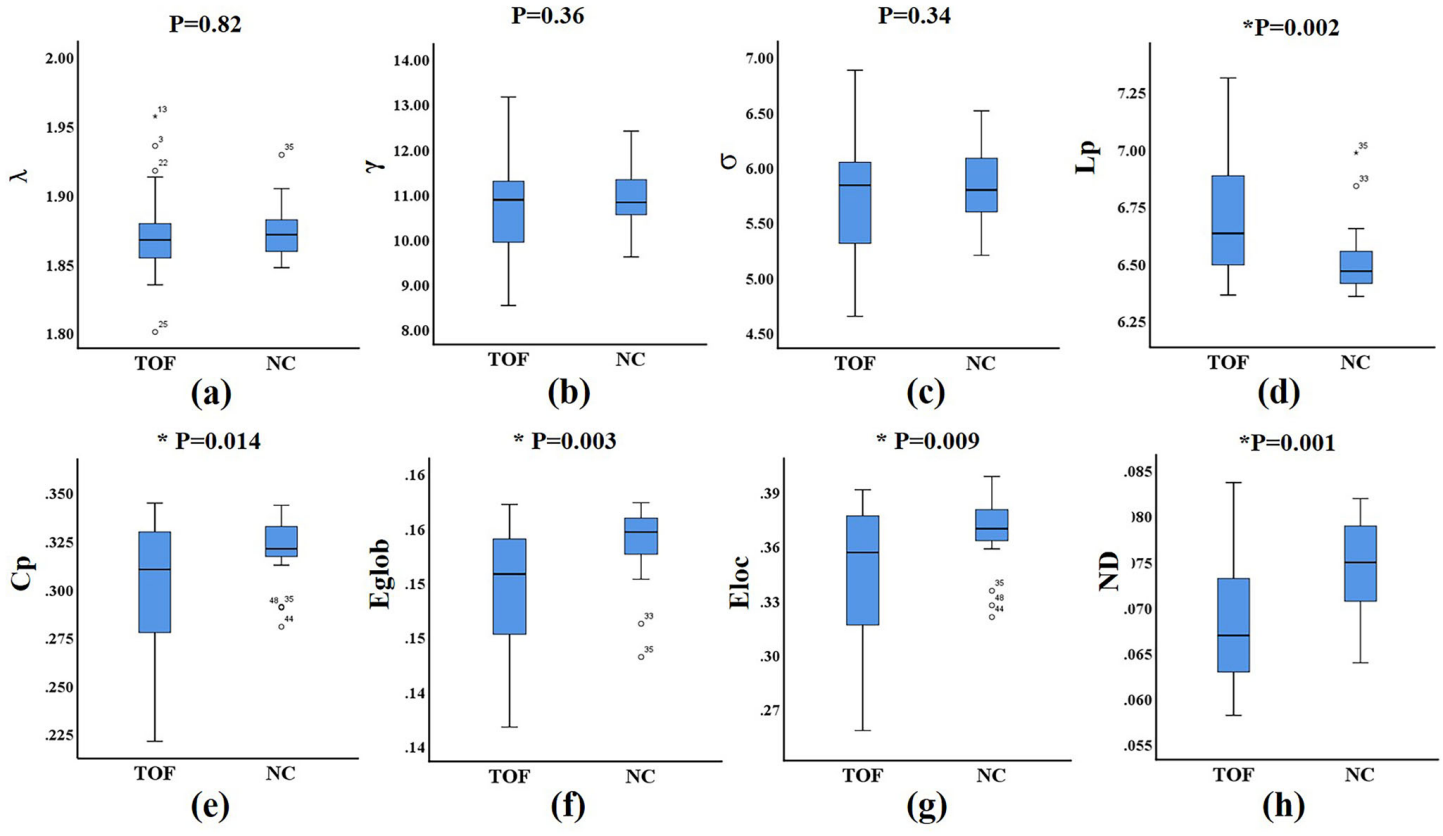
Global properties of the structural brain network in TOF patients and controls. There were no significant differences in small‐worldness between the two groups **(a‐c)**. Cp, Eglob, Eloc and ND were decreased, while Lp was increased in TOF compared with controls **(d‐g)**. Cp, clustering coefficient; Lp, characteristic path length; Eglob, global efficiency; Eloc, local efficiency; ND, network density; γ, normalized clustering coefficient; λ, normalized characteristic path length; σ = γ/λ, small‐worldness.

Figure 2 and Table 2 illustrate the global properties of the structural brain network in TOF and controls. Both children with TOF and non‐CHD exhibited small‐worldness organization, and there were no significant differences in small‐worldness between the two groups. Additionally, in comparison with controls, TOF patients demonstrated decreased values in Cp (*t* = 2.56, *p* = 0.014), Eglob (*t* = 3.13, *p* = 0.003), Eloc (*t* = 2.76, *p* = 0.009), and ND (*t* = 3.72, *p* = 0.001), while Lp (*t* = ‐3.20, *p* = 0.002) exhibited an increase.

**TABLE 2:** The difference in global properties of the structural brain network of the structural network between children with TOF and non‐CHD.

	TOF	Non‐CHD	*t*	*p*
Λ	1.87 ± 0.03	1.87 ± 0.02	0.23	0.82
γ	10.72 ± 1.07	10.95 ± 0.66	0.92	0.36
σ	5.72 ± 0.53	5.84 ± 0.34	0.96	0.34
Lp	6.72 ± 0.28	6.51 ± 0.16	−3.20	0.002
Cp	0.30 ± 0.03	0.32 ± 0.02	2.56	0.014
Eglob	0.15 ± 0.01	0.15 ± 0.00	3.13	0.003
Eloc	0.34 ± 0.04	0.37 ± 0.02	2.76	0.009
ND	0.68 ± 0.01	0.75 ± 0.00	3.72	0.001

*Note*: λ, normalized Lp (Lp real/Lp rand); γ, normalized Cp (Cp real/Cp rand); σ, small‐worldness (γ/λ); Cp, clustering coefficient; Lp, characteristic path length; Eglob, global efficiency; Eloc, local efficiency; ND, network density.

**FIGURE 3:**
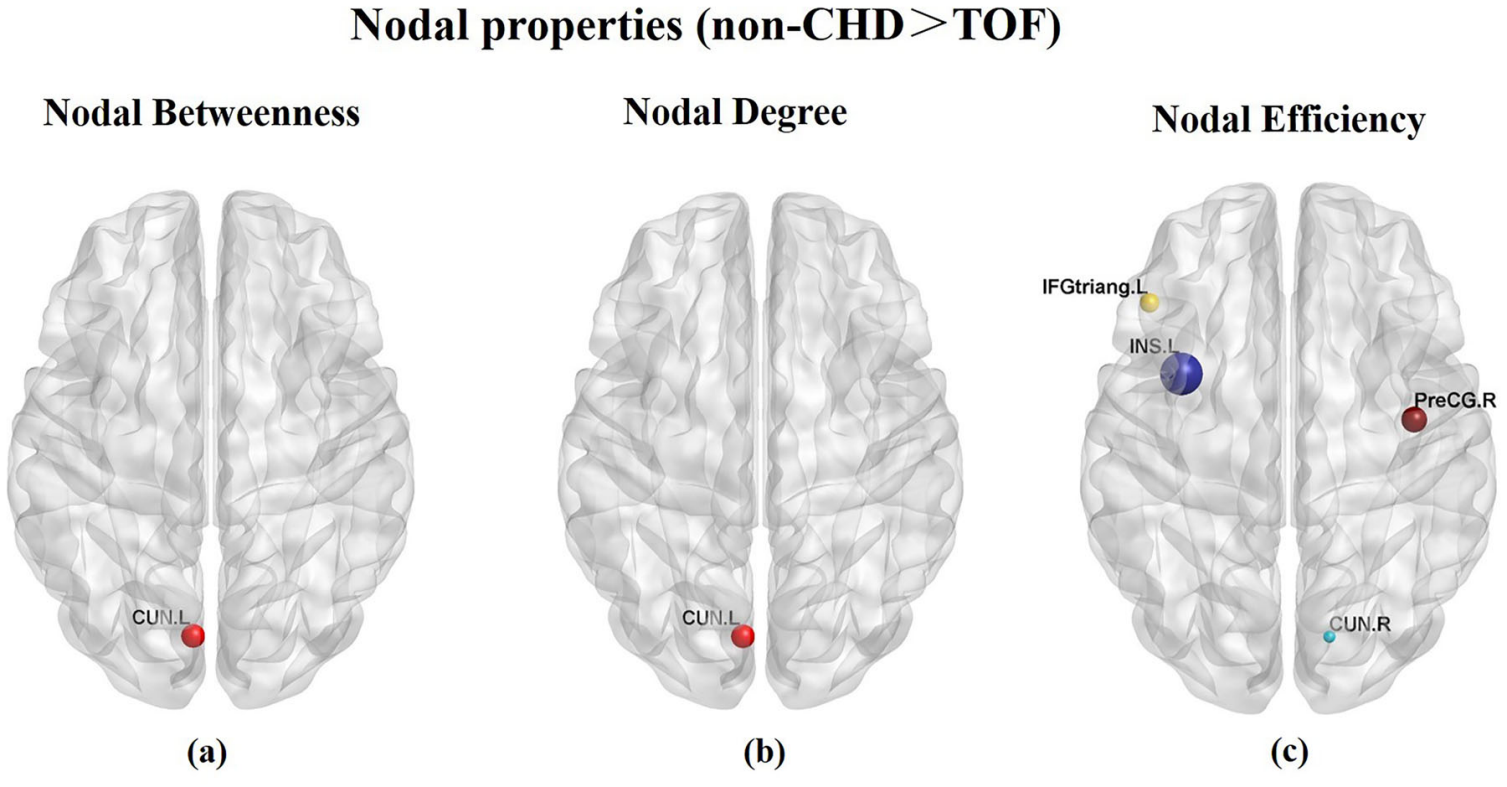
Compared with controls, TOF groups exhibited decreased nodal betweenness and degree in the left cingulate gyrus **(a‐b)** and decreased nodal efficiency in the right precentral gyrus, left inferior frontal gyrus (triangular part), left insula and right cingulate gyrus **(c)**.

Compared with the controls, the TOF groups exhibited decreased nodal betweenness and degree in the left cingulate gyrus (*t* = 2.78, *p* = 0.007, *t* = 2.99, *p* = 0.004) and decreased nodal efficiency in the right precentral gyrus (*t* = 3.12, *p* = 0.003), left inferior frontal gyrus (triangular part) (*t* = 3.35, *p* = 0.002), left insula (*t* = 3.27, *p* = 0.002), and right cingulate gyrus (*t* = 3.27, *p* = 0.002). Multiple comparison correction was performed using false‐positive correction. However, there was no significant difference in nodal properties after FDR correction. Nodal parameters of the structural brain network after false‐positive correction are shown in Table 3 and Figure 3.

**TABLE 3:** The difference in nodal parameters of the structural network between TOF and non‐CHD.

Parameters	Regions	TOF	Non‐CHD	*t*	*p*
Nodal betweenness	Left cingulate gyrus	171.29 ± 27.47	190.99 ± 21.05	2.78	0.007
Nodal degree	Left cingulate gyrus	6.17 ± 0.85	6.74 ± 0.45	2.99	0.004
Nodal efficiency	Right precentral gyrus	0.19 ± 0.01	0.19 ± 0.00	3.12	0.003
	Left inferior frontal gyrus	0.18 ± 0.00	0.18 ± 0.00	3.35	0.002
	Left insular	0.22 ± 0.01	0.23 ± 0.00	3.27	0.002
	Right cingulate gyrus	0.17 ± 0.01	0.17 ± 0.00	3.27	0.002

Abbreviations: CHD, congenital heart disease; TOF, tetralogy of fallot.

### 3.3 Relationship between Clinical Data and Topology Parameters and Cognitive Performance

**FIGURE 4:**
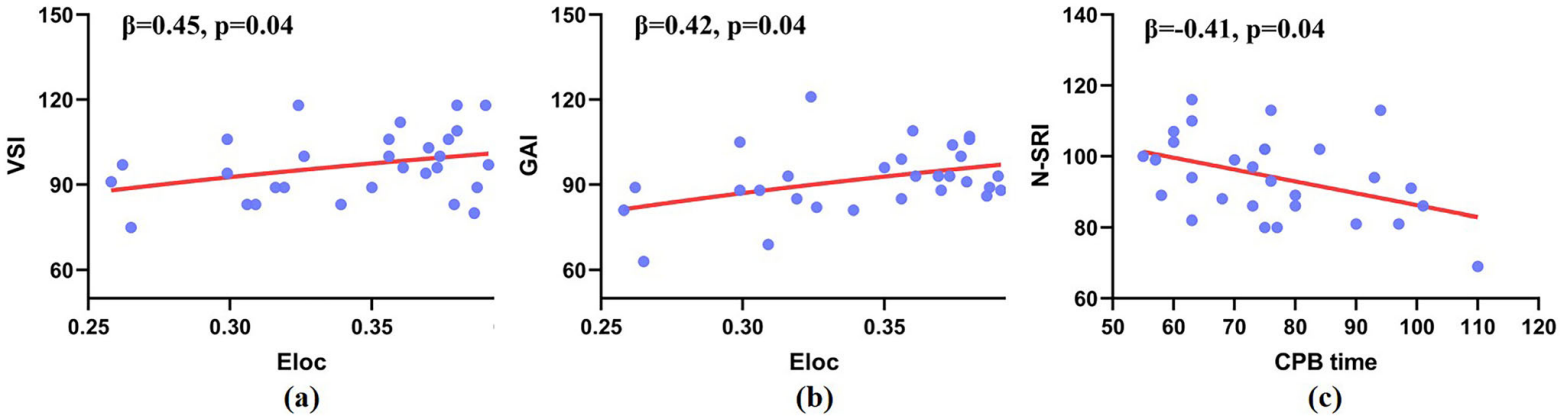
TOF group showed a significantly positive correlation between Eloc and VSI as well as GAI **(a, b)** A significantly negative correlation between N‐SRI and CPB time was also found in TOF group **(c)** CPB, cardiopulmonary bypass; Eloc, local efficiency; GAI, general ability index; N‐SRI, non‐speech reception index; VSI, visual spatial index.

The TOF group showed a significantly positive correlation between Eloc and VSI (*p* = 0.04, *B* = 0.45) as well as GAI (*p* = 0.04, *B* = 0.42), shown in Figure 4 and Supplement Table 2. Additionally, a significantly negative correlation between N‐SRI and CPB time (*p* = 0.04, *B* = −0.41) was identified within the TOF group, as illustrated in Supplement Table 1.

## 4 Discussion

In this study, we investigated brain structural network topology by constructing a white matter network through DTI tractography in TOF. We found revealed that (1) both TOF and non‐CHD groups exhibited small‐world networks. However, children with TOF showed increased Lp, decreased Cp, network density, Eglob, and Eloc when compared with non‐CHD. (2) Aberrant nodal properties in the precentral gyrus, inferior frontal gyrus, insular, and cingulate gyrus were observed in the TOF group; these findings may imply a worse white matter network topological organization in the TOF group, consistent with prior research (Panigrahy et al. 2015; Schmithorst et al. 2018). Furthermore, local efficiency correlated with cognitive performance in TOF, offering novel insights into comprehending the mechanisms underlying cognitive deficits associated with TOF based on brain structural connectivity.

### 4.1 Whole Brain Topology Properties

Human brain information processing entails two fundamental aspects: integration and segregation. Integration involves the amalgamation of processed information from various brain regions, which is subsequently relayed back to the central brain center. Segregation, on the other hand, entails the division of tasks into distinct components, allocated to corresponding functional regions (Albert, Jeong, and Barabasi 2000). The human brain can be considered a network characterized by high‐efficiency integration and segregation named the small‐world network (Rubinov and Sporns 2010; Bullmore and Sporns 2009). Small‐world networks have high Cp and low Lp, which is a measure of the balance between integration and segregation (Fair et al. 2007). In this study, both children with TOF and non‐CHD displayed small‐world network topology, consistent with prior research (Panigrahy et al. 2015; Schmithorst et al. 2018), suggesting that the brain still maintains the balance between integration and segregation in TOF. However, there was no statistically significant disparity in small‐world topology between the two groups, contrary to previous findings (Panigrahy et al. 2015). We speculate that this discrepancy may stem from variations in CHD subtypes and associated anatomical and physiological alterations.

Lp serves as a metric for assessing information transmission capacity, reflecting the long‐distance connectivity characteristics of the network (Watts and Strogatz 1998). The observed increase Lp in this study implies a reduction in the network's long‐distance connectivity and information transmission capacity, indicative of diminished global network features. Consequently, the transmission and integration of long‐distance information among different functional regions may be impaired in children with TOF. A previous study based on DTI tractography proposed that degeneration of white matter fibers for information transmission may lead to increased Lp (Bai et al. 2012). We speculated that abnormal Lp observed in children with TOF may indeed be attributed to white matter fiber degeneration, warranting further confirmation in future studies. Cp often represents functional specialization or segregation, serving as an indicator of the local information‐processing capacity within a network. Decreased Cp suggested that local information processing and network segregation may be damaged in children with TOF. Consistent with our findings, a previous study demonstrated that the integration and separation features of brain structural connections were disrupted in neonates with CHD (Feldmann et al. 2020). However, the aforementioned study focused on mixed‐type CHD, where anatomical, physiological, and hemodynamic stability can influence brain development (Peyvandi et al. 2019). Our study directly reflects changes in brain network organization in specific CHD subtypes compared to their broader investigation.

Network density, defined as the average of the total number of connections, serves as an index of network connectivity compactness and is utilized to evaluate the comprehensive integration across brain regions (Danielle et al. 2017). The reduced network density observed in children with TOF in this study may suggest a weakening of the connectivity of brain regions in children with TOF. TOF is a complex congenital heart disease that may affect brain development through multiple mechanisms. A previous study found decreased network density and functional connectivity during brain development in children under anesthesia (Desowska, Berde, and Cornelissen 2023). We speculated that chronic hypoxia, abnormal blood circulation, or stress responses associated with surgery may adversely affect the formation and maintenance of brain networks (Bonthrone et al. 2022).

Eglob represents the capacity of information transmission over the network and can measure the rate of information transmission (Rubinov and Sporns 2010). It is closely associated with long‐range connectivity and the integration of brain networks, facilitating the rapid amalgamation of information across distinct neural regions. In this study, decreased Eglob suggests a diminished efficiency of information transmission within the global network and impaired global information integration in children with TOF. In line with our study, Schmithorst et al. noted that Eglob reduced in networks based on different weighted and unweighted approaches in neonates with CHD (Schmithorst et al. 2018). Although Panigrahy et al. also reported decreased Eglob in a TGA, it was only at a trend level without significant difference between the two groups (Panigrahy et al. 2015). This disparity may be attributed to differences in CHD subtypes.

Eloc represents the capacity of information transmission at the local level and measures the fault tolerance of the network (Achard and Bullmore 2007). Decreased Eloc indicated reduced efficiency of information transmission in local regions and that communication is vulnerable to mistakes in children with TOF. Previous studies have highlighted significant alterations in the topological properties of the brain structural network in CHD, potentially leading to cognitive impairments such as deficits in memory, executive function, and visual spatial function (Panigrahy et al. 2015). In line with these findings, our study revealed a positive correlation between Eloc and cognitive performance, including verbal comprehension index (VSI) and general ability index (GAI), suggesting that aberrant network efficiency is associated with cognitive deficits. Furthermore, we observed a negative correlation between cardiopulmonary bypass (CPB) time and neurobehavioral outcomes, indicating that shorter CPB durations during surgery may contribute to cognitive protection.

### 4.2 Regional Node Properties

Regional analysis can elucidate and characterize specific nodal dysfunction in children with TOF. In this study, we observed decreased nodal betweenness and degree in the left cingulate gyrus, along with reduced nodal efficiency in the right precentral gyrus, left inferior frontal gyrus (triangular part), left insula, and right cingulate gyrus in TOF. Previous research has documented delayed cortical development across multiple brain regions, including the cingulate gyrus, inferior frontal region, superior temporal gyrus, and postcentral gyrus, in fetuses with hypoplastic left heart syndrome (Clouchoux et al. 2013). Additionally, DTI investigations have identified microstructural abnormalities in various areas, such as the frontal regions, precentral gyrus, insula, and hippocampus, with reduced fractional anisotropy (FA) correlating with deficits in executive function and attention (Rivkin et al. 2013; Rollins et al. 2014). The regions exhibiting decreased nodal parameters in our study overlap with those reported previously, suggesting that structural abnormalities in these brain areas may underlie changes in nodal properties and diminished efficiency in information transformation. A recent investigation also reported decreased nodal efficiency in the frontal, insular, limbic, and occipital lobes (Feldmann et al. 2020), further aligning with our findings. Overall, the similarity between structural abnormalities in CHD and our findings in preschool TOF implies that nodal properties could serve as predictive indicators of brain structural alterations in CHD and that such abnormalities may persist into the preschool period, reflecting a continuum of delayed brain development.

### 4.3 Limitations

There are several limitations in this study. Firstly, our cross‐sectional study did not investigate the difference in the brain structural network between preoperative and postoperative in children with TOF, and we have not yet conducted genetic‐related testing for these children. Future research aims to recruit children with TOF who have undergone genetic testing, both before and after surgery, to further explore the impact of surgery on the brain after excluding genetic factors. Secondly, a statistical comparison of cognitive performance between non‐CHD and TOF patients is lacking due to some non‐CHD failing to complete the WPPSI‐IV because of the long time needed. Future endeavors involve continued recruitment of children with TOF while concurrently collecting scale data from typically developing children without congenital heart disease. Thirdly, normalization and construction of the brain network were executed using an adult template due to the absence of a standardized brain template for preschool children. Fourthly, the structural network was constructed solely based on FA, not including MD and tract numbers. Previous studies have demonstrated that graph analyses based on FA are more sensitive to microstructural changes than those based on other parameters (Schmithorst et al. 2018). Lastly, since the sample size of this study was small, FDR or Bonferroni correction was too severe, and the results were not significant, we chose false‐positive correction. The same correction method was also used in Lynall's (Lynall et al. 2010) and Meng's (Meng et al. 2014) neuroimage study, which may not avoid Class I errors. Large‐scale studies in the future are warranted to validate our research findings.

## 5 Conclusion

This study revealed disrupted brain structural network topology in preschool children with TOF, characterized by impaired integration and segregation, which correlates with cognitive performance. These findings underscore the significance of early neuroprotective interventions aimed at enhancing brain development and cognitive function to mitigate lasting impairments throughout the lifespan. Furthermore, the correlation observed between brain network topology properties and cognitive performance suggests that cognitive deficits in patients may partly stem from disrupted brain structural connectivity in TOF.

## Author Contributions

**Yuting Liu**: writing–original draft, data curation, formal analysis, methodology. **Liang Hu**: resources, investigation. **Meijiao Zhu**: writing–review and editing, validation. **Jingjing Zhong**: visualization, investigation. **Mingcui Fu**: supervision. **Mingwen Yang**: validation. **Shuting Cheng**: validation. **Ying Wang**: visualization. **Xuming Mo**: conceptualization, methodology, project administration, resources, supervision. **Ming Yang**: supervision, methodology, project administration.

## Conflicts of Interest

The authors report no disclosures relevant to the manuscript.

### Peer Review

The peer review history for this article is available at https://publons.com/publon/10.1002/brb3.70153

## Ethics Statement

All procedures performed in studies involving human participants were approved by the Institutional Ethics Committee of Children's Hospital of Nanjing Medical University. Written informed consent was offered by the guardian of each subject.

## Supporting information



Supplement Table 1 Linear regression results of clinical data and cognitive performance in children with TOF.
Supplement Table 2 Linear regression results of brain network topological parameters and cognitive performance in children with TOF.

## Data Availability

The dataset used and analyzed are available to other researchers subject to review of the request by the Scientific Committee of the study and ethical approval.

## References

[brb370153-bib-0001] Achard, S. , and E. Bullmore . 2007. “Efficiency and Cost of Economical Brain Functional Networks.” PLoS Computational Biology 3, no. 2: e17. 17274684 10.1371/journal.pcbi.0030017 PMC1794324

[brb370153-bib-0002] Albert, R. , H. Jeong , and A. L. Barabasi . 2000. “Error and Attack Tolerance of Complex Networks.” Nature 406, no. 6794: 378–382. 10935628 10.1038/35019019

[brb370153-bib-0003] Bai, F. , N. Shu , Y. Yuan , et al. 2012. “Topologically Convergent and Divergent Structural Connectivity Patterns Between Patients with Remitted Geriatric Depression and Amnestic Mild Cognitive Impairment.” The Journal of Neuroscience: The Official Journal of the Society for Neuroscience 32, no. 12: 4307–4318. 22442092 10.1523/JNEUROSCI.5061-11.2012 PMC6621223

[brb370153-bib-0004] Bonthrone, A. , R. Stegeman , M. Feldmann , et al. 2022. “Risk Factors for Perioperative Brain Lesions in Infants with Congenital Heart Disease: A European Collaboration.” Stroke; A Journal of Cerebral Circulation 53, no. 12: 3652–3661. 10.1161/STROKEAHA.122.039492 PMC9698124 36300371

[brb370153-bib-0005] Bullmore, E. , and O. Sporns . 2009. “Complex Brain Networks: Graph Theoretical Analysis of Structural and Functional Systems.” Nature Reviews Neuroscience 10, no. 3: 186–198. 19190637 10.1038/nrn2575

[brb370153-bib-0006] Clouchoux, C. , A. J. du Plessis , M. Bouyssi‐Kobar , et al. 2013. “Delayed Cortical Development in Fetuses with Complex Congenital Heart Disease.” Cerebral Cortex 23, no. 12: 2932–2943. 22977063 10.1093/cercor/bhs281

[brb370153-bib-0007] Cui, Z. , S. Zhong , P. Xu , Y. He , and G. Gong . 2013. “PANDA: A Pipeline Toolbox for Analyzing Brain Diffusion Images.” Frontiers in Human Neuroscience 7: 42. 23439846 10.3389/fnhum.2013.00042 PMC3578208

[brb370153-bib-0008] Danielle, C. F. , Z. M. Asim , E. B. Andrew , B. M. Mark , K. Bang Bon , and J. K. Ronald . 2017. “Retained Executive Abilities in Mild Cognitive Impairment Are Associated with Increased White Matter Network Connectivity.” European Radiology 28, no. 1: 340–347. 28695358 10.1007/s00330-017-4951-4 PMC5798454

[brb370153-bib-0009] de Asis‐Cruz, J. , M. T. Donofrio , G. Vezina , and C. Limperopoulos . 2018. “Aberrant Brain Functional Connectivity in Newborns with Congenital Heart Disease Before Cardiac Surgery.” NeuroImage Clinical 17: 31–42. 29034164 10.1016/j.nicl.2017.09.020 PMC5635248

[brb370153-bib-0010] Desowska, A. , C. Berde , and L. Cornelissen . 2023. “Emerging Functional Connectivity Patterns During Sevoflurane Anaesthesia in the Developing Human Brain.” British Journal of Anaesthesia 130, no. 2: e381–e90. 10.1016/j.bja.2022.05.033 35803755

[brb370153-bib-0011] Easson, K. , C. V. Rohlicek , J. C. Houde , et al. 2020. “Quantification of Apparent Axon Density and Orientation Dispersion in the White Matter of Youth Born with Congenital Heart Disease.” Neuroimage 205: 116255. 31605826 10.1016/j.neuroimage.2019.116255

[brb370153-bib-0012] Fair, D. A. , N. U. Dosenbach , J. A. Church , et al. 2007. “Development of Distinct Control Networks Through Segregation and Integration.” Proceedings of the National Academy of Sciences of the United States of America 104, no. 33: 13507–13512. 17679691 10.1073/pnas.0705843104 PMC1940033

[brb370153-bib-0013] Feldmann, M. , T. Guo , S. P. Miller , et al. 2020. “Delayed Maturation of the Structural Brain Connectome in Neonates with Congenital Heart Disease.” Brain Communications 2, no. 2: fcaa209. 33381759 10.1093/braincomms/fcaa209 PMC7756099

[brb370153-bib-0014] Fontes, K. , C. V. Rohlicek , C. Saint‐Martin , et al. 2019. “Hippocampal Alterations and Functional Correlates in Adolescents and Young Adults with Congenital Heart Disease.” Human Brain Mapping 40, no. 12: 3548–3560. 31070841 10.1002/hbm.24615 PMC6865495

[brb370153-bib-0015] Jane, M. W. , J. E. Karen , G. Linda , et al. 2019. “Neurodevelopmental and Health‐Related Quality‐of‐Life Outcomes in Adolescence After Surgery for Congenital Heart Disease in Infancy.” Developmental Medicine and Child Neurology 62, no. 2: 214–220. 31025336 10.1111/dmcn.14251

[brb370153-bib-0016] Kaku, S. M. , A. Jayashankar , S. C. Girimaji , S. Bansal , S. Gohel , and R. D. Bharath . 2019. “Srinath S. Early Childhood Network Alterations in Severe Autism.” Asian Journal of Psychiatry 39: 114–119. 30610990 10.1016/j.ajp.2018.12.009

[brb370153-bib-0017] Kelly, C. J. , A. Makropoulos , L. Cordero‐Grande , et al. 2017. “Impaired Development of the Cerebral Cortex in Infants with Congenital Heart Disease Is Correlated to Reduced Cerebral Oxygen Delivery.” Scientific Reports 7, no. 1: 15088. 29118365 10.1038/s41598-017-14939-z PMC5678433

[brb370153-bib-0018] Lynall, M. E. , D. S. Bassett , R. Kerwin , et al. 2010. “Functional Connectivity and Brain Networks in Schizophrenia.” The Journal of Neuroscience: The Official Journal of the Society for Neuroscience 30, no. 28: 9477–9487. 20631176 10.1523/JNEUROSCI.0333-10.2010 PMC2914251

[brb370153-bib-0019] Meng, C. , F. Brandl , M. Tahmasian , et al. 2014. “Aberrant Topology of Striatum's Connectivity Is Associated with the Number of Episodes in Depression.” Brain: A Journal of Neurology 137, no. Pt 2: 598–609. 24163276 10.1093/brain/awt290

[brb370153-bib-0020] Morton, S. U. , L. Maleyeff , D. Wypij , et al. 2020. “Abnormal Left‐Hemispheric Sulcal Patterns Correlate with Neurodevelopmental Outcomes in Subjects with Single Ventricular Congenital Heart Disease.” Cerebral Cortex 30, no. 2: 476–487. 31216004 10.1093/cercor/bhz101 PMC7306172

[brb370153-bib-0021] Mulkey, S. , X. Ou , R. Ramakrishnaiah , et al. 2014. “White Matter Injury in Newborns with Congenital Heart Disease: A Diffusion Tensor Imaging Study.” Pediatric Neurology 51, no. 3: 377–383. 25160542 10.1016/j.pediatrneurol.2014.04.008 PMC4147255

[brb370153-bib-0022] Naef, N. , R. Liamlahi , I. Beck , et al. 2017. “Neurodevelopmental Profiles of Children with Congenital Heart Disease at School Age.” The Journal of Pediatrics 188: 75–81. 28709631 10.1016/j.jpeds.2017.05.073

[brb370153-bib-0023] Olsen, M. , H. T. Sorensen , V. E. Hjortdal , T. D. Christensen , and L. Pedersen . 2011. “Congenital Heart Defects and Developmental and Other Psychiatric Disorders: A Danish Nationwide Cohort Study.” Circulation 124, no. 16: 1706–1712. 21947292 10.1161/CIRCULATIONAHA.110.002832

[brb370153-bib-0024] Owen, J. P. , E. Ziv , P. Bukshpun , et al. 2013. “Test‐Retest Reliability of Computational Network Measurements Derived from the Structural Connectome of the Human Brain.” Brain Connectivity 3, no. 2: 160–176. 23350832 10.1089/brain.2012.0121 PMC3634151

[brb370153-bib-0025] Panigrahy, A. , V. J. Schmithorst , J. L. Wisnowski , et al. 2015. “Relationship of White Matter Network Topology and Cognitive Outcome in Adolescents with D‐Transposition of the Great Arteries.” NeuroImage Clinical 7: 438–448. 25685710 10.1016/j.nicl.2015.01.013 PMC4318874

[brb370153-bib-0026] Petersen, S. E. , and O. Sporns . 2015. “Brain Networks and Cognitive Architectures.” Neuron 88, no. 1: 207–219. 26447582 10.1016/j.neuron.2015.09.027 PMC4598639

[brb370153-bib-0027] Peyvandi, S. , B. Latal , S. P. Miller , and P. S. McQuillen . 2019. “The Neonatal Brain in Critical Congenital Heart Disease: Insights and Future Directions.” Neuroimage 185: 776–782. 29787864 10.1016/j.neuroimage.2018.05.045

[brb370153-bib-0028] Rivkin, M. J. , C. G. Watson , L. A. Scoppettuolo , et al. 2013. “Adolescents with D‐Transposition of the Great Arteries Repaired in Early Infancy Demonstrate Reduced White Matter Microstructure Associated with Clinical Risk Factors.” The Journal of Thoracic and Cardiovascular Surgery 146, no. 3: 543–549.e1. 23375991 10.1016/j.jtcvs.2012.12.006 PMC3748216

[brb370153-bib-0029] Rollins, C. K. , C. G. Watson , L. A. Asaro , et al. 2014. “White Matter Microstructure and Cognition in Adolescents with Congenital Heart Disease.” The Journal of Pediatrics 165, no. 5: 936–944.e1–2. 25217200 10.1016/j.jpeds.2014.07.028 PMC4258111

[brb370153-bib-0030] Rubinov, M. , and O. Sporns . 2010. “Complex Network Measures of Brain Connectivity: Uses and Interpretations.” Neuroimage 52, no. 3: 1059–1069. 19819337 10.1016/j.neuroimage.2009.10.003

[brb370153-bib-0031] Sa de Almeida, J. , D. E. Meskaldji , S. Loukas , et al. 2021. “Preterm Birth Leads to Impaired Rich‐Club Organization and Fronto‐Paralimbic/Limbic Structural Connectivity in Newborns.” Neuroimage 225: 117440. 33039621 10.1016/j.neuroimage.2020.117440

[brb370153-bib-0032] Sananes, R. , C. Manlhiot , E. Kelly , et al. 2012. “Neurodevelopmental Outcomes after Open Heart Operations Before 3 Months of Age.” The Annals of Thoracic Surgery 93, no. 5: 1577–1583. 22541188 10.1016/j.athoracsur.2012.02.011

[brb370153-bib-0033] Schmithorst, V. J. , J. K. Votava‐Smith , N. Tran , et al. 2018. “Structural Network Topology Correlates of Microstructural Brain Dysmaturation in Term Infants with Congenital Heart Disease.” Human Brain Mapping 39, no. 11: 4593–4610. 30076775 10.1002/hbm.24308 PMC6260793

[brb370153-bib-0034] Shu, N. , Y. Liu , K. Li , et al. 2011. “Diffusion Tensor Tractography Reveals Disrupted Topological Efficiency in White Matter Structural Networks in Multiple Sclerosis.” Cerebral Cortex 21, no. 11: 2565–2577. 21467209 10.1093/cercor/bhr039

[brb370153-bib-0035] Sidlauskaite, J. , K. Caeyenberghs , E. Sonuga‐Barke , H. Roeyers , and J. R. Wiersema . 2015. “Whole‐Brain Structural Topology in Adult Attention‐Deficit/Hyperactivity Disorder: Preserved Global—Disturbed Local Network Organization.” NeuroImage Clinical 9: 506–512. 26640763 10.1016/j.nicl.2015.10.001 PMC4630025

[brb370153-bib-0036] Tabbutt, S. , J. W. Gaynor , and J. W. Newburger . 2012. “Neurodevelopmental Outcomes after Congenital Heart Surgery and Strategies for Improvement.” Current Opinion in Cardiology 27, no. 2: 82–91. 22274574 10.1097/HCO.0b013e328350197b

[brb370153-bib-0037] Uehara, T. , T. Yamasaki , T. Okamoto , et al. 2014. “Efficiency of a “Small‐World” Brain Network Depends on Consciousness Level: A Resting‐State FMRI Study.” Cerebral Cortex 24, no. 6: 1529–1539. 23349223 10.1093/cercor/bht004

[brb370153-bib-0038] van der Linde, D. , E. E. Konings , M. A. Slager , et al. 2011. “Birth Prevalence of Congenital Heart Disease Worldwide: A Systematic Review and Meta‐Analysis.” Journal of the American College of Cardiology 58, no. 21: 2241–2247. 22078432 10.1016/j.jacc.2011.08.025

[brb370153-bib-0039] Watts, D. J. , and S. H. Strogatz . 1998. “Collective Dynamics of ‘Small‐World’ Networks.” Nature 393, no. 6684: 440–442. 9623998 10.1038/30918

[brb370153-bib-0040] Yagi, Y. , M. Yamamoto , H. Saito , et al. 2017. “Changes of Cerebral Oxygenation in Sequential Glenn and Fontan Procedures in the Same Children.” Pediatric Cardiology 38, no. 6: 1215–1219. 28589407 10.1007/s00246-017-1647-0

[brb370153-bib-0041] Yang, M. , Y. Liu , S. Ma , et al. 2022. “Altered Brain Structure in Preschool‐Aged Children with Tetralogy of Fallot.” Pediatric Research 93, no. 5: 1321–1327. 35194163 10.1038/s41390-022-01987-z

[brb370153-bib-0042] Yuan, W. , S. Wade , and L. Babcock . 2015. “Structural Connectivity Abnormality in Children with Acute Mild Traumatic Brain Injury Using Graph Theoretical Analysis.” Human Brain Mapping 36, no. 2: 779–792. 25363671 10.1002/hbm.22664 PMC5500248

