# Supplementary material for: Disrupted White Matter Topology Organization in Preschool Children with Tetralogy of Fallot
Source: Brain Behav. 2024 Nov 22;14(11):e70153. doi: 10.1002/brb3.70153 (PMC11583477; doi:10.1002/brb3.70153)
Supplement: Supplementary file 1 — Supplement Table 1 Linear regression results of clinical data and cognitive performance in children with TOF. Supplement Table 2 Linear regression results of brain network topological parameters and cognitive performance in children with TOF. [file BRB3-14-e70153-s001.docx]

Supplement Table1 Linear regression results of clinical data and cognitive performance in children with TOF

|  | VCI | | VSI | | WMI | | FSIQ | | SRI | | N-SRI | | GAI | |
| --- | --- | --- | --- | --- | --- | --- | --- | --- | --- | --- | --- | --- | --- | --- |
|  | B | p | B | p | B | p | B | p | B | p | B | p | B | p |
| age of surgery | -0.07 | 0.72 | -0.24 | 0.29 | -0.16 | 0.44 | -0.19 | 0.38 | -0.21 | 0.25 | -0.10 | 0.61 | -0.23 | 0.19 |
| hospitalization day | -0.08 | 0.62 | -0.36 | 0.06 | 0.08 | 0.63 | -0.09 | 0.64 | -0.21 | 0.25 | -0.10 | 0.61 | -0.23 | 0.19 |
| operational time | 0.10 | 0.56 | -0.01 | 0.96 | -0.21 | 0.23 | -0.12 | 0.56 | 0.09 | 0.64 | -0.13 | 0.52 | 0.13 | 0.50 |
| CPB time | -0.11 | 0.53 | -0.39 | 0.06 | -0.18 | 0.32 | 0.36 | 0.06 | -0.23 | 0.21 | -0.41 | 0.04 | -0.17 | 0.36 |
| ACC time | -0.04 | 0.83 | -0.28 | 0.01 | 0.97 | 0.64 | -0.12 | 0.51 | -0.13 | 0.50 | -0.07 | 0.65 | -0.08 | 0.66 |

Note: CPB, cardiopulmonary bypass; ACC, aortic cross-clamp; VCI, verbal comprehension index; VSI, visual spatial index; WMI, working memory index; FSIQ, full-scale intelligence quotient; SRI, speech reception index; N-SRI, non-speech reception index; GAI, general ability index.

Supplement Table2 Linear regression results of brain network topological parameters and cognitive performance in children with TOF

|  | VCI | | VSI | | WMI | | FSIQ | | SRI | | N-SRI | | GAI | |
| --- | --- | --- | --- | --- | --- | --- | --- | --- | --- | --- | --- | --- | --- | --- |
|  | B | p | B | p | B | p | B | p | B | p | B | p | B | p |
| λ | 0.13 | 0.47 | 0.33 | 0.13 | -0.14 | 0.46 | 0.17 | 0.37 | 0.09 | 0.57 | 0.16 | 0.45 | 0.31 | 0.11 |
| δ | 0.02 | 0.91 | 0.26 | 0.19 | 0.15 | 0.38 | 0.16 | 0.39 | -0.02 | 0.88 | 0.29 | 0.15 | 0.16 | 0.38 |
| γ | 0.03 | 0.87 | 0.28 | 0.15 | 0.12 | 0.50 | 0.17 | 0.37 | -0.01 | 0.93 | 0.28 | 0.15 | 0.19 | 0.30 |
| Lp | -0.37 | 0.07 | -0.25 | 0.31 | -0.16 | 0.46 | -0.39 | 0.08 | -0.17 | 0.38 | -0.26 | 0.29 | 0.34 | 0.13 |
| Cp | 0.24 | 0.19 | 0.41 | 0.07 | 0.16 | 0.41 | 0.33 | 0.10 | 0.08 | 0.63 | 0.35 | 0.10 | 0.38 | 0.06 |
| Eglob | 0.34 | 0.08 | 0.25 | 0.34 | 0.17 | 0.44 | 0.38 | 0.09 | 0.16 | 0.43 | 0.26 | 0.28 | 0.32 | 0.16 |
| Eloc | 0.28 | 0.14 | 0.45 | 0.04 | 0.18 | 0.36 | 0.38 | 0.06 | 0.09 | 0.61 | 0.38 | 0.08 | 0.42 | 0.04 |
| ND | -0.46 | 0.45 | 0.67 | 0.29 | 0.10 | 0.87 | 1.92 | 0.07 | 0.27 | 0.54 | -0.84 | 0.22 | -1.49 | 0.14 |

Note: λ, normalized Lp (Lp real/Lp rand); γ, normalized Cp (Cp real/Cp rand); δ, small-worldness ( γ/λ); Cp, clustering coefficient; Lp, path length; Eglob, global efficiency; Eloc, local efficiency; ND, network density; VCI, verbal comprehension index; VSI, visual spatial index; WMI, working memory index; FSIQ, full-scale intelligence quotient; SRI, speech reception index; N-SRI, non-speech reception index; GAI, general ability index.
